# Identification of *Campylobacter jejuni* and *Chlamydia psittaci* from cockatiel (*Nymphicus hollandicus*) using metagenomics

**DOI:** 10.1186/s12864-021-08122-y

**Published:** 2021-11-06

**Authors:** Si-Hyeon Kim, Yong-Kuk Kwon, Choi-Kyu Park, Hye-Ryoung Kim

**Affiliations:** 1 Avian Disease Division, Animal and Plant Quarantine Agency, 177 Hyeoksin 8-ro, Gyeongsangbuk-do 39660 Gimcheon-si, Republic of Korea; 2 Animal Disease Intervention Center, College of Veterinary Medicine, Kyungpook National University, 41566 Daegu, Republic of Korea

**Keywords:** Pet birds, Cockatiel, Metagenomics, Chlamydiosis, Campylobacteriosis, Recombination, MetaSPAdes

## Abstract

**Background:**

In July 2015, the carcasses of 11 cockatiels were submitted for disease diagnosis to the Avian Disease Division of the Animal and Plant Quarantine Agency of Korea. The cockatiels, which appeared dehydrated and underweight, had exhibited severe diarrhea and 22 % mortality over 2 weeks. Traditional diagnosis did not reveal the causes of these symptoms.

**Methods:**

We conducted metagenomics analysis on intestines and livers from the dead cockatiels using Illumina high-throughput sequencing. To obtain more accurate and longer contigs, which are required for further genetic characterization, we compared the results of three *de novo* assembly tools (metaSPAdes, MEGAHIT, and IDBA-UD).

**Results:**

Sequence reads of *Campylobacter jejuni* (*C. jejuni*) and *Chlamydia psittaci* (*C. psittaci*) were present in most of the cockatiel samples. Either of these bacteria could cause the reported symptoms in psittaciformes. metaSPAdes (ver.3.14.1) identified the 1152 bp *flaA* gene of *C. jejuni* and the 1096 bp *ompA* gene of *C. psittaci*. Genetic analysis revealed that *flaA* of *C. jejuni* was recombinant between *C. jejuni* and *Campylobacter coli*, and that *ompA* of *C. psittaci* isolated from cockatiel was closely related to strains isolated from humans.

**Conclusions:**

*C. jejuni* and *C. psittaci* were detected in cockatiels in the Republic of Korea using metagenomic analysis. This approach is useful for understanding pathogens of pet birds. Three *de novo* assemblers were compared to obtain accurate contigs from large quantities of reads, and sequences of *C. jejuni* and *C. psittaci* generated by metaSPAdes were analyzed.

## Background

Transmittable diseases of parrots include pulmonary diseases such as psittacosis, influenza, histoplasmosis, Newcastle disease, Q fever, and West Nile virus fever; gastrointestinal diseases such as salmonellosis, campylobacteriosis, and giardiasis; and cutaneous diseases such as pasteurellosis, erysipeloid cryptococcosis, mite dermatitis, and nontuberculous mycobacteriosis [1]. In addition, a few viral diseases, such as proventricular dilation disease caused by avian bornavirus, psittacine beak and feather disease caused by circovirus, and polyomavirus infection (also called budgerigar fledgling disease and Pacheco’s disease) caused by psittacid herpesvirus 1, are frequently lethal to pet birds [2–5]. Many pet bird diseases are asymptomatic but can be transmitted to humans via inhalation or ingestion of infected or contaminated material [1]. *Campylobacter*, *Salmonella*, and *Arcobacter* have been detected in apparently healthy pet birds, indicating that these animals are potential carriers of these enteropathogens to humans [6]. Zoonotic transmission of disease from psittacid species is uncommon but still represents a public health danger [7]. Therefore, disease diagnosis and pathogen identification in parrots are important for public health and potential to human-infection pathogens.

**Fig. 1:**
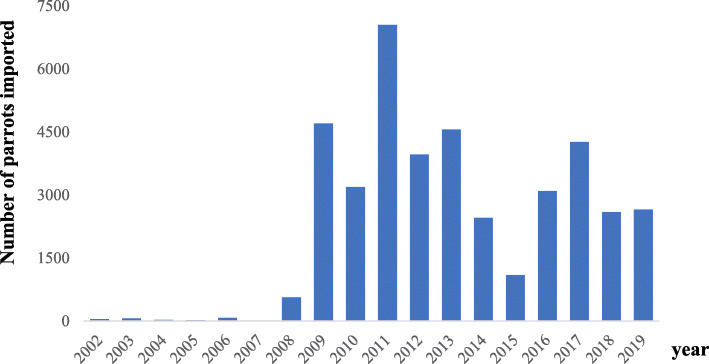
Number of imported parrots approved by the Animal and Plant Quarantine Agency of the Republic of Korea between 2002 and 2019 (http://eminwon.qia.go.kr/statistics)

No parrot species are native to the Republic of Korea, but import of pet parrots has recently increased (Fig. 1) despite bans on unregulated trafficking. Meanwhile, the pet bird industry, including breeding facilities, parrot shops, and pet bird owners, has grown exponentially. Cockatiels (*Nymphicus hollandicus*) are popular pet parrots around the world because they are gregarious, small, and elegantly colored; moreover, their propagation in captivity is relatively simple. Several studies have described diseases of cockatiels and the fecal microbiomes of healthy cockatiels [8–10], but information on cockatiel diseases is limited relative to the body of knowledge on other birds such as poultry.

Traditional diagnostic methods, such as microorganism culture, nucleic acid amplification tests, and serologic assay, which require a great deal of time and labour, and have limitations for finding the etiology of infectious disease [11]. Recent advances in high-throughput sequencing technologies have been used for pathogen detection and discovery [12]. Metagenomics, which can reveal a high degree of microbial diversity, can be used to determine the etiology of diseases with unknown causes [13–15].

In this study, we conducted metagenomics analysis to elucidate the cause of diarrhea in cockatiels that had not been revealed by traditional diagnosis. In addition, we compared the results of three *de novo* assemblers to obtain accurate sequences of *Campylobacter jejuni* (*C. jejuni*) and *Chlamydia psittaci* (*C. psittaci*), and then performed genetic characterizations on these sequences.

## Methods

### Samples and purification

In July 2015, the carcasses of 11 cockatiels were submitted for disease diagnosis to the Avian Disease Division of the Animal and Plant Quarantine Agency (APQA). The 30-day-old birds appeared dehydrated and underweight, and had exhibited severe diarrhea and 22 % mortality over the course of 2 weeks. The dead cockatiels were shipped the next day refrigerated and inspected. On the basis of clinical manifestations and the presence of gross lesions, necropsy, bacteriological culture using three culture media per sample, defibrinated sheep blood agar plate, MacConkey agar plate and LB broth, virus isolation using specific pathogen-free embryonated chicken eggs, pathological examination, and electron microscopy were performed according to an APQA diagnostic protocol. Each of the 11 cockatiels had been bred in the same place and had similar symptoms and gross lesions, so 4 samples were chosen at random for metagenomic analysis. To identify the pathogen, the intestines (ileum and colon) and livers of four cockatiels (*15AD75*-*1* to *15AD75-4*) were collected after necropsy, pooled for each individual, and promptly processed via blending into a 10 % homogenate in sterile phosphate-buffered saline (PBS) containing 0.4 mg/mL gentamicin. Healthy chicken trachea samples were used as negative control for the sample pretreatment and sequencing. The homogenates were centrifuged at 3,500 r.p.m. and 13,000 r.p.m. for 10 min each. To remove large particles, the supernatants of intestine homogenates were filtered using 0.8 and 0.45 μm syringe filters. To eliminate free DNA, viral particles were pelleted by ultracentrifugation (30,000 r.p.m., 5 h, 4 °C), resuspended in 500 µL of 1 M Tris-Cl (pH 7.4), and treated with 2.5 units of DNase I (AMPD1; Sigma-Aldrich, St. Louis, MO, USA) for 3 h at 37 °C. The sample was concentrated and washed twice using a Microcon 30 column (Millipore, USA). DNase activity was inhibited by addition of 0.5 M EDTA to a final concentration of 20 mM.

### High-throughput sequencing

The homogenates were immediately frozen and kept at -80 ℃ until RNA extraction. Total RNA was extracted from purified samples using the Viral Gene-spin Viral DNA/RNA Extraction Kit (iNtRON Biotechnology, Republic of Korea). cDNA synthesis and PCR amplification of nucleic acid were carried out in a 50 µL mixture containing 5 µg RNA, 0.5 µM random primer K (GAC CAT CTA GCG ACC TCC AC), and 0.5 µM primer KN (GAC CAT CTA GCG ACC TCC CAN NNN NNN N) as described previously [16] using the Access RT-PCR system (Promega, USA). The products were purified using an UltraClean PCR Clean-up Kit (MO BIO, USA) and sequenced at Theragen Etex (Suwon, Republic of Korea). Sample libraries were prepared using the Illumina TruSeq DNA sample preparation kit (Illumina, USA), and DNA was fragmented using a Covaris adaptive focused acoustics device to generate double-stranded DNA fragments 300–400 bp in size. The ends were repaired, phosphorylated, and 3’-end adenylated.

Paired-end DNA adaptors (Illumina) were ligated, and construct fragments of ~500 bp were selected. Libraries were loaded onto a paired-end flow cell and sequenced as 101 bp paired-end, indexed reads on an Illumina HiSeq 2500 instrument. The raw read sequences were filtered using the following exclusion criteria: (1) presence of ambiguous bases (letter N) in excess of 10 %; (2) average quality below 20; (3) more than 5 % of nucleotides with quality below 20; or (4) the presence of an adapter sequence. Total reads were aligned with the NCBI RefSeq viral, bacterial, and fungal genome database using the Burrows–Wheeler Aligner software (ver.0.7.12). To annotate the reads, homology-based (BLAST) classification (ver.2.2.31) based on the nucleotide sequence was performed using the NCBI ‘nucleotide’ database. Taxonomy was determined using in-house script based on the NCBI taxonomy database (blastn e-value cutoff < e^−450^ ) [17–19].

### Sequence analysis

To obtain more accurate assembled sequences, we compared the results of three assembly tools, IDBA-UD (ver.1.1.1) [20], MEGAHIT (ver.1.2.9) [21], and metaSPAdes (ver.3.14.1) [22]. The three assemblers were run using 96 threads (Intel® Xeon® Platinum 8260 Processor, 2CPU). MEGAHIT used the least peak memory (average 3.77 Gb) and had the shortest runtime (average 1.07 h). By contrast, IDBA-UD and metaSPAdes required higher memory consumption and longer times (55.41 Gb and 3.25 h; 44.6 Gb and 3.39 h, respectively). Quality of contigs was analyzed by MetaQUAST (ver.5.0.2) misassemblies are defined by the following criteria (a) the left flanking sequence aligns over 1 kb away from the right flanking sequence on the reference (b) flanking sequences overlap on more than 1 kb (c) flanking sequences align to different strands or different chromosomes (d) flanking sequences align on different reference genomes; mismatches per 100 kb are defined by the following criteria (a) the average number of mismatches per 100,000 aligned bases (b) True SNPs and sequencing errors are not distinguished and are counted equally; indels per 100 kb is the average number of indels per 100,000 aligned bases. Several consecutive single nucleotide indels are counted as one indel [23]. With minimum alignment of 65 bp and 95–100 % identity. Sequences were extracted using Seqs-Extractor (ver.1.1.2). RDP (ver.4.1.01) was used for detection and analysis of recombination [24]. Sequences were aligned and analyzed using the CLC main workbench 7 (CLC Bio, Denmark). Phylogenetic trees were generated by the neighbor-joining method with 1,000 bootstrap replications using the MEGA 10 software [25] on related species published in the DDBJ/EMBL/GenBank database.

## Results

### Disease diagnosis of cockatiels

The only gross lesion in the dead cockatiels was weak fibrinous perihepatitis. *Escherichia coli* was identified in the liver by bacteriological culture. The presence of avian bornavirus, avian polyomavirus, beak and feather disease virus, and psittacid herpesvirus 1 was investigated by PCR, but none of these viruses were detected. Hemagglutination test on harvested allantoic fluid was negative. Microscopic lesions were observed in liver multifocal hepatitis, perivascular lymphocyte and heterophil infiltration, minor pericarditis, and lung congestion.

### Metagenomics analysis

Four cockatiel samples (*15AD75-1*, *-2*, *-3*, and *-4*) were sequenced, yielding 8,160,360 to 14,041,743 reads each. All reads of four cockatiel samples and control were broadly classified into one of four groups: (1) eukaryotic (95.2–99.9 %), (2) bacterial (<0.1–1.7 %), (3) viral (<0.1 %), and (4) unidentified (<0.1–3.9 %). Unidentified reads may not be coding region because sequencing of conserved neighboring genes improves taxonomic assignment when analyzing metagenomes using Sanger sequencing [26]. Homology-based (BLAST) classification of 1,614–144,494 bacterial reads identified four different bacterial species with sequence identity to avian bacteria. The identified bacterial sequences were assigned to known families, including *C. jejuni* (*Campylobacteraceae*), *C. psittaci* (*Chlamydiaceae*), *Escherichia coli* (*Enterobacteriaceae*), and *Clostridium colinum*. *C*. *jejuni* was detected in all four samples, and *C. psittaci* sequences were detected in three (*15AD75-1*, *-2*, and *-3*). Sequence reads classified as *Enterobacteriaceae* were identified in sample *15AD75-2*, and *C. coli* sequences were identified in sample *15AD75-1*. Although the sample preparation enriched for viruses, viral sequence reads represented fewer than 0.1 % of reads in three samples and were completely absent in the other sample, *15AD75-4* (Table 1). *C. jejuni* and *C. psittaci* were detected in three or four samples. Because these two organisms are pathogens in psittaciformes, we analyzed both in detail.

**Table 1:** Number of reads assigned using BLAST with cockatiel samples and control sample

	*15AD75-1*	*15AD75-2*	*15AD75-3*	*15AD75-4*	*Control*
Total reads	8,842,628	8,160,360	8,503,438	14,041,743	18,929,276
Eukaryota (% total reads)	8,420,210 (95.2)	8,148,391 (99.9)	8,354,957 (98.3)	13,951,068 (99.4)	18,839,539 (99.5)
Bacteria (% total reads)	76,253 (0.9)	1,614 (<0.1)	144,494 (1.7)	80,294 (0.9)	57,770 (0.3)
*Chlamydia psittaci* (% bacteria)	51,147 (67.1)	109 (6.8)	142,521 (98.6)	-	-
*Campylobacter jejuni* (% bacteria)	24,072 (31.6)	1,387 (85.9)	1,158 (0.8)	78,015 (97.2)	-
*Enterobacteriaceae*	-	32	-	-	2,823
*Clostridium colinum*	149	-	-	-	-
*Salmonella enterica*	-	-	-	-	19,573
*Raoultella ornithinolytica*	-	-	-	-	14,815
*Citrobacter sp.*	-	-	-	-	10,401
*Escherichia coli*	-	-	-	-	6,158
*Methylophaga sp.*	-	-	-	-	1,179
*Lysinibacillus sphaericus*	-	-	-	-	1,045
etc.	-	-	-	-	1,776
unassigned	885	118	815	2,279	3
Virus (% total reads)	2,582 (<0.1)	3,530 (<0.1)	809 (<0.1)	-	13,145 (<0.1)
*retroviridae*	2,514	3,420	752	-	-
hepatitis B virus	4	-	57	-	-
herpesvirus	-	110	-	-	-
Escherichia phage	-	-	-	-	11,275
Enterobacteria phage	-	-	-	-	1,617
Streptococcus phage	-	-	-	-	222
Rous sarcoma virus	-	-	-	-	31
Unidentified (% total reads)	343,083 (3.9)	6,825 (0.1)	3,178 (<0.1)	10,381 (0.1)	31,967 (0.17)

### Comparison of the ***de novo*** assembly tools

To obtain more accurate and longer sequence contigs of bacteria, we compared the results of three *de novo* assemblers (IDBA-UD, MEGAHIT, and metaSPAdes) using reads classified as *C. jejuni* from sample *15AD75-1* and reads classified as *C. psittaci* from sample *15AD75-3*. The most accurate assembly was generated by metaSPAdes with 0 misassemblies, 1383.12 mismatches per 100 kb, and 15.4 indels per 100 kb in *C. jejuni.* Moreover, metaSPAdes had only one misassembly with 40.68 mismatches per 100 kb and 8.62 indel per 100 kb in *C. psittaci*. The most inaccurate assembly was generated by MEGAHIT. In *C. jejuni*, MEGAHIT yielded slightly longer contigs, with N50 of 681, largest alignment of 3,004, and genome fraction of 3.84 %, but had the most misassemblies (4). Similarly, in *C. psittaci*, MEGAHIT yielded slightly longer contigs, with N50 of 1,104, largest alignment of 5,732, and genome fraction of 61.90, but also had most misassemblies (13). IDBA-UD had intermediate accuracy, with no misassemblies in *C. jejuni* but 11 in *C. psittaci* (Table 2).

**Table 2:** Statistics of *Campylobacter jejuni* and *Chlamydia psittaci* contigs compared with results of three assembly tools using MetaQUAST

	*Campylobacter jejuni*			*Chlamydia psittaci*		
assembler	IDBA-UD	MEGAHIT	metaSPAdes	IDBA-UD	MEGAHIT	metaSPAdes
N50	669	**681**	630	1,057	**1,104**	974
longest contigs (bp)	1,938	**3,004**	2,880	4,842	**5,732**	5,398
genome fraction (%)	2.76	**3.84**	2.95	52.75	**61.90**	56.41
misassemblies	**0**	4	**0**	11	13	**1**
mismatches per 100 kb	1,462.90	1,395.01	**1,383.12**	**39.13**	45.75	40.68
indels per 100 kb	20.71	18.83	**15.40**	**3.23**	3.72	8.62

*Campylobacter jejuni 15AD75-1* contigs were compared to reference *Campylobacter jejuni ZJB021* (accession no. CP048767.1) and *Chlamydia psittaci 15AD75-3* contigs to *Chlamydia psittaci GIMC 2005* (accession no. CP024451.1)

The best value for each column is indicated in bold

**Fig. 2:**
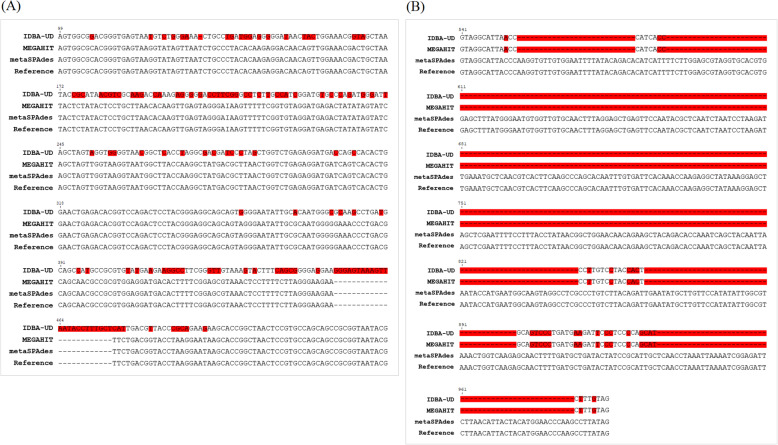
Comparison of contigs generated by alignment sequence using IDBA-UD, MEGAHIT, and metaSPAdes assembly tools. **A** Comparison of the 16srRNA gene of *Campylobacter jejuni 15AD75-1* (GenBank accession no. MW534394) with that of *C. jejuni ZJB021* (GenBank accession no. CP048767.1) **B** Comparison of the *ompA* gene of *Chlamydia psittaci 15AD75-3* (GenBank accession no. MW544064) with that of *C. psittaci GIMC 2005* (GenBank accession no. CP024451.1). Red boxes indicate misassembled regions

Next, using sample *15AD75-1*, we compared C. *jejuni* 16S rRNA gene sequences, generated by IDBA-UD, MEGAHIT, and metaSPAdes assembly tools, with the reference sequence of *C. jejuni ZJB021* (GenBank accession no. CP048767.1). The sequence aligned by IDBA-UD had a 28 nt insertion at positions 451–478 (GGGAGTAAAGTTAATACCTTTGCTCAT) instead of TTC, as well as various misassemblies, but sequences obtained using MEGAHIT and metaSPAdes had no misassemblies. MEGAHIT and metaSPAdes showed 100 % nt identity with the 16srRNA gene of *Campylobacter jejuni ZJB021* (accession no. CP048767.1) but IDBA-UD only had 74 % nt identity (Fig. 2 A). We then compared *ompA* sequences of *C. psittaci* from sample *15AD75-3*, generated by IDBA-UD, MEGAHIT, and metaSPAdes, with the reference sequence *C. psittaci GIMC 2005* (GenBank accession no. CP024451.1). Sequences aligned by IDBA-UD and MEGAHIT had many misassemblies at positions 541–994 and consisted of short contigs (615 nt), but the sequence obtained using metaSPAdes had zero misassemblies and was 994 nt in length. MetaSPAdes showed 100 % nt identity with the ompA gene of *Chlamydia psittaci 15AD75-3* (GenBank accession no. MW544064) but IDBA-UD and MEGAHIT only provided 59 % nt identity (Fig. 2B). Thus, more accurate and longer sequences were generated by metaSPAdes. These sequences were deposited in the GenBank database under accession numbers MW534394 and MW544064.

### Genetic analysis

**Fig. 3:**
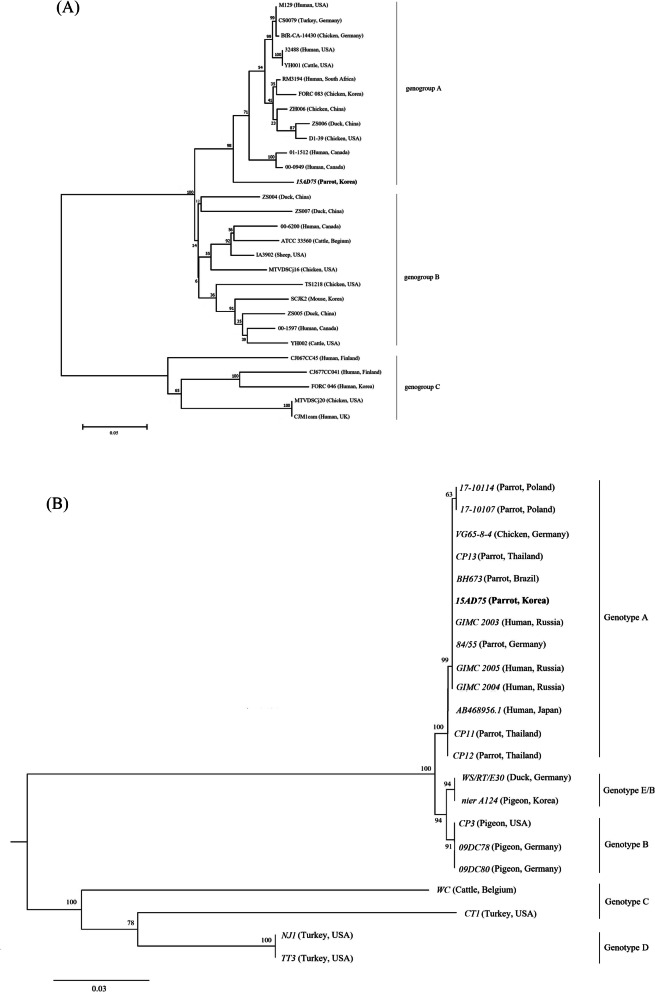
Phylogenetic analysis of nucleotide sequences of (**A**) *flaA* of *Campylobacter jejuni* and (**B**) *ompA* of *Chlamydia psittaci*, generated by the neighbor-joining method using MEGA X with 1,000 bootstrap replications. Strains identified in this study are indicated in bold

**Fig. 4:**
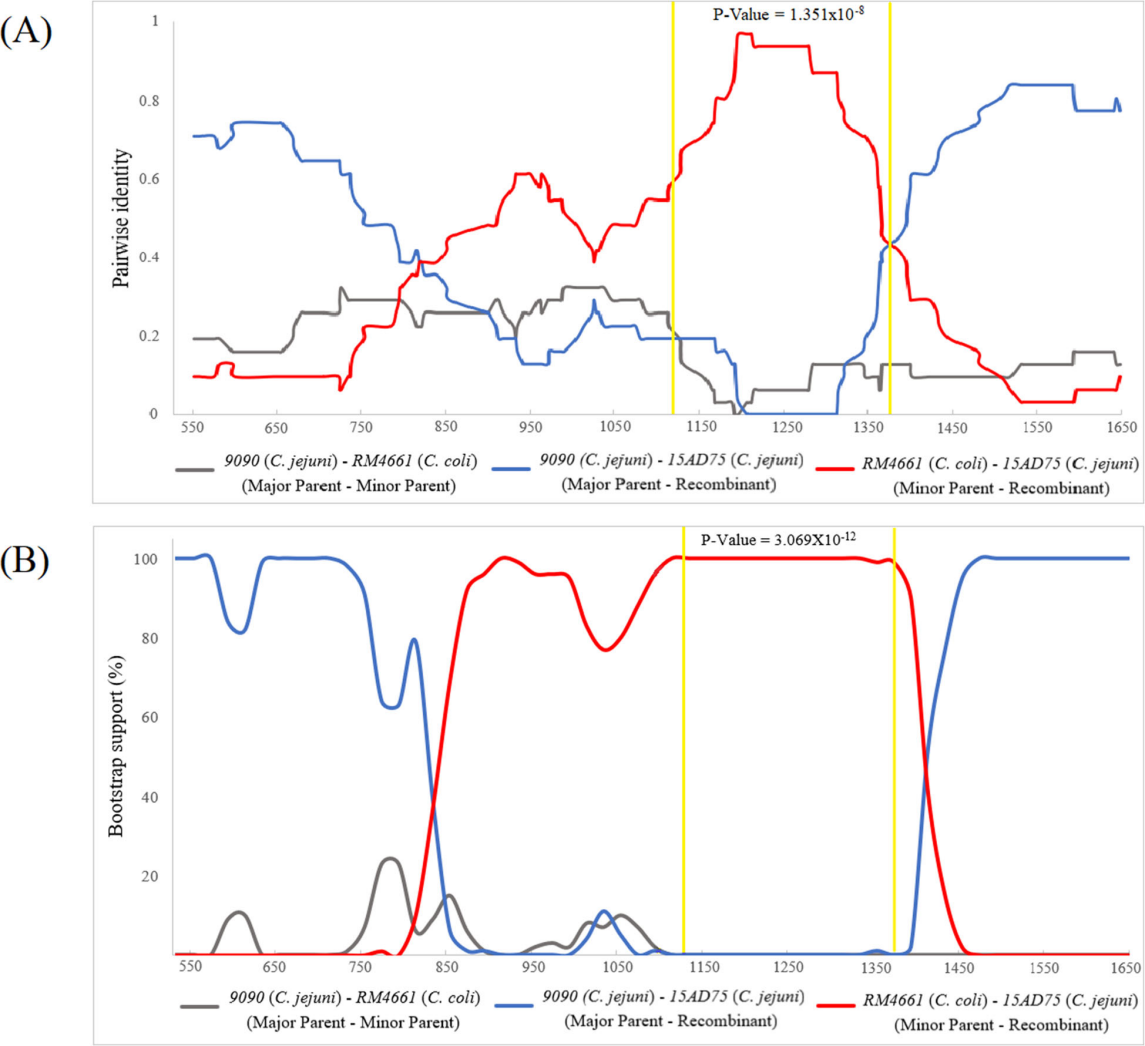
Pairwise identity plot (**A**) and bootstrap support plot (**B**) achieved by multiple sequence alignments of *flaA* of *Campylobacter* spp. using Recombination Detection Program (RDP4). *flaA* of strain *15AD75* (*C. jejuni*) was recombinant, sharing homology with strain *9090* (*C. jejuni*) and RM4661 (*C. coli*)

To genetically characterize the identified bacteria relative to known references, we generated phylogenetic trees of the *flaA* gene of C. *jejuni* and the *ompA* gene of *C. psittaci* using the neighbor-joining method. Figure 3A shows three clusters of *flaA* sequences that were not correlated with host or country. C. *jejuni 15AD75* was closely related to a cluster in genogroup A but was not sub-grouped with other strains. This partial *flaA* gene (1152 bp) of *15AD75* (accession no. MW544065) had 89.61 % nucleotide identity to strain C. *jejuni 9090* (accession no. CP007181.1), but also had 91.23 % nucleotide identity to strain *C. coli RM4661* (accession no. CP007181.1). In the recombination analysis using RDP, we detected a significant recombination event between breakpoints (positions 1121 and 1377), with *C. coli RM4661* (Turkey, USA) as the minor parent (*P*-value = 1.351 × 10^-8^) and *C. jejuni 9090* (Human, Slovenia) as the major parent (Fig. 4A). Recombination was supported by bootstrap support with a P-value of 3.609 × 10^-12^ (Fig. 4B). *15AD75* had 97.6 % nucleotide sequence identity with *Campylobacter coli* (*C. coli*) *RM4661* (accession no. CP007181.1) and 75.1 % identity with *C. jejuni 9090* (accession no. CP007181.1) at breakpoints. The breakpoint sequences were closer to *C. coli* than to C. *jejuni*.

In the phylogram, *ompA* of the *C. psittaci* was clustered in genotype A, the major genotype associated with strains from parrots, chicken, and humans (Fig. 3B). *C. psittaci 15AD75* was similar to reference parrot strains (accession no. MH507065.1, MH507064.1, KR010621.1, MH138297.1, CP003790.1, KR010619.1, KR010620.1), with more than 99.89 % sequence identity. In addition, it was similar to human isolates, with 100 % and 99.87 % identity to strains from Russia (accession no. CP024453.1, CP024451.1, CP024455.1) and Japan (accession no. AB468956.1), respectively.

## Discussion

The efficiencies of next-generation sequencing platforms such as Ion Torrent, Illumina, and Nanopore have been compared for metagenomic sequencing [27, 28]. Metagenomic sequencing-based approaches to disease diagnosis have the potential to overcome the shortcomings of both culture and PCR [29, 30]. Metagenomic analysis has a few limitations. Assuming the same depth of sequencing, samples with an abundance of non-pathogen RNA show lower analytical sensitivity than samples in which pathogen RNA is more abundant. The more reads that are sequenced, the further the sensitivity but also cost increases [31, 32]. The sensitivity limitations could be overcome with a host depletion method, which would reduce the proportion of host being sequenced, leading to better coverage over pathogens without having to sequence at higher depth. Few studies have compared bioinformatics tools devoted to tasks such as *de novo* assembly and annotation. When we use metagenomics for diagnosis of disease, both accurate sequencing and identification of pathogen genes are necessary to analyze gene characteristics, as culture and isolation processes are not available. In this study, we found that metaSPAdes generated more accurate assemblies than MEGAHIT or IDBA-UD when using the same fastq data, but required a great deal of additional computational resources and processing time. MEGAHIT is a *de novo* assembler designed for assembly of large and complex metagenomics data in a time- and cost-efficient manner [21]. Therefore, we recommended using metaSPAdes to achieve the most accurate results, and MEGAHIT when it is necessary to minimize cost or processing time.

Using metagenomics analysis, we identified two bacteria, *C. jejuni* and *C. psittaci*, from sick cockatiels, that both pathogens were not cultured and identified in the traditional method, and obtained accurate long sequence reads using the metaSPAdes assembly tool. So, metagenomic can be used for pathogen detection beyond the shortcomings of culture. Additional genetic analysis of sequences from both bacteria revealed that the *C. jejuni* identified in this study was recombinant with *C. coli* and *C. psittaci* had strong homology with strains isolated from humans.

*C. jejuni* is an enteric organism that is well adapted to avian hosts, and most *Campylobacter* infections produce minimal or no disease. However, consumption of poultry meat, which has a high prevalence of *Campylobacter*, is a significant cause of human food poisoning [33, 34]. Previously, *Campylobacter* had not been detected in the normal microbiota of psittacines such as cockatiels and scarlet macaws [9, 35, 36], and some studies reported that *C. jejuni* causes diarrhea in young chicks, turkey poults, and Japanese quail [34]. In addition, recombination of the *flaA* gene can increase antigenic diversity, allowing the bacterium to escape the immunological responses of the host [37]. Some variants of *C. jejuni* have a greater capacity to survive environmental pressures and colonize the host gut [38]. Accordingly, this species could have been responsible for the diarrhea observed in the sick cockatiels examined in this study.

Order Psittaciformes contains the species that are most susceptible to chlamydial infection, which causes economic losses in both the pet bird and poultry industries, and represents a public health threat to people in close contact with infected birds [39, 40]. Clinically, chlamydial infections in most avian species produce severe airsacculitis and pneumonitis, although pharyngeal specimens from many healthy cockatiels have high titers of chlamydia [41]. Because the cockatiels tested in this study had digestive diseases such as diarrhea and perihepatitis, but not respiratory diseases, the *C. psittaci* identified by our metagenomics analysis might have been involved in subclinical infections.

Identification of *C. jejuni* and *C. psittaci* in pet birds is significant for public health. *Campylobacter* has emerged as a leading cause of foodborne gastroenteritis in humans worldwide [42]. In addition, *C. jejuni* invades tissue and induces inflammation, resulting in the post-infection autoimmune disease Guillain–Barré syndrome [43]. Many types of birds can be infected by *C. psittaci*. Domestic and companion birds are considered the main risk for transmission of psittacosis to humans [44]. The US Centers for Disease Control and Prevention reported 935 human cases of psittacosis from 1988 to 2003, most of which were related to contact with Psittaciformes [45]. Therefore, a surveillance program to control diseases of pet birds, including *C. psittaci* infections, as well as pet bird hygiene and biosecurity management of all bird-related transactions, are needed as soon as possible to decrease the prevalence of these infections.

## Conclusions

*C. jejuni* and *C. psittaci* were detected for the first time by metagenomic analysis in cockatiels in the Republic of Korea. The findings of this study are useful for understanding pet bird pathogens. Three *de novo* assemblers were compared to obtain accurate contigs from large quantities of reads, and the sequences of *C. jejuni* and *C. psittaci* generated by metaSPAdes were analyzed.

## Data Availability

The reference genomes used during the current study are publicly available and were downloaded from the NCBI nucleotide database (https://www.ncbi.nlm.nih.gov/nuccore). BWA is available at (https://github.com/lh3/bwa) under the GPLv3 License. BLAST is available at (https://blast.ncbi.nlm.nih.gov/Blast.cgi?CMD=Web&PAGE_TYPE=BlastDocs&DOC_TYPE=Download). IDBA-UD is available at (https://github.com/loneknightpy/idba) under the GPLv2 License. MEGAHIT is available at (https://github.com/voutcn/megahit) under the GPLv3 License. metaSPAdes is available at (https://github.com/ablab/spades) under the GPLv2 License. The DNA-sequencing data were deposited to Sequence Read Archive (SRA) under accession number PRJNA756392.

## Abbreviations

APQA: Animal and Plant Quarantine Agency
BLAST: Basic Local Alignment Search Tool
*C. coli*: *Campylobacter coli*
*C. jejuni*: *Campylobacter jejuni*
*C. psittaci*: *Chlamydia psittaci*
kb: Kilobase
nt: Nucleotide
RDP: Recombination Detection Program
